# Effects of Arousal on Mouse Sensory Cortex Depend on Modality

**DOI:** 10.1016/j.celrep.2018.02.092

**Published:** 2018-03-20

**Authors:** Daisuke Shimaoka, Kenneth D. Harris, Matteo Carandini

**Affiliations:** 1 UCL Institute of Ophthalmology, University College London, Gower Street, London WC1E 6AE, UK; 2 UCL Institute of Neurology, University College London, Gower Street, London WC1E 6AE, UK; 3 Department of Neuroscience, Physiology and Pharmacology, University College London, Gower Street, London WC1E 6AE, UK

**Keywords:** cerebral cortex, cortical state, locomotion, sensory processing, widefield imaging

## Abstract

Changes in arousal modulate the activity of mouse sensory cortex, but studies in different mice and different sensory areas disagree on whether this modulation enhances or suppresses activity. We measured this modulation simultaneously in multiple cortical areas by imaging mice expressing voltage-sensitive fluorescent proteins (VSFP). VSFP imaging estimates local membrane potential across large portions of cortex. We used temporal filters to predict local potential from running speed or from pupil dilation, two measures of arousal. The filters provided good fits and revealed that the effects of arousal depend on modality. In the primary visual cortex (V1) and auditory cortex (Au), arousal caused depolarization followed by hyperpolarization. In the barrel cortex (S1b) and a secondary visual area (LM), it caused only hyperpolarization. In all areas, nonetheless, arousal reduced the phasic responses to trains of sensory stimuli. These results demonstrate diverse effects of arousal across sensory cortex but similar effects on sensory responses.

## Highlights

• Voltage-sensitive fluorescent proteins reveal effects of arousal across the cortex
• In auditory and primary visual areas, arousal depolarizes then hyperpolarizes
• In somatosensory and secondary visual areas, arousal only hyperpolarizes
• In all areas, arousal reduces phasic responses to trains of sensory stimuli

## Graphical Abstract

**Figure:**
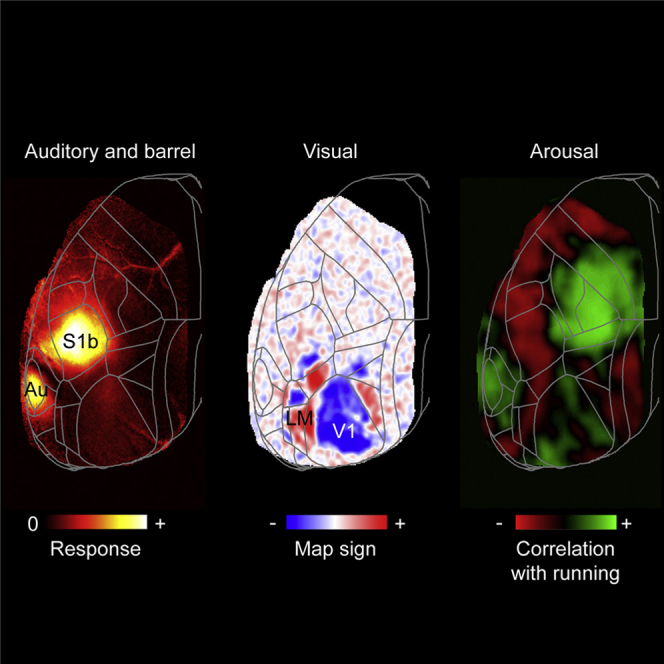

## Teaser

Shimaoka et al. use voltage-sensitive imaging to show that the effects of arousal on the mouse cortex are markedly different across areas and over time. In all the sensory areas studied, nonetheless, arousal reduced the phasic voltage responses to trains of sensory stimuli.

## Introduction

There is increasing evidence that arousal has widespread effects on the activity of the cerebral cortex, providing a powerful nonsensory modulation even to primary sensory areas such as the primary visual cortex (V1) and primary auditory cortex (A1). Much of this evidence has been obtained in mice, where changes in arousal can be measured from pupil dilations (McGinley et al., 2015a, McGinley et al., 2015b, Vinck et al., 2015) and locomotion (Ayaz et al., 2013, Bennett et al., 2013, Erisken et al., 2014, Fu et al., 2014, Lee et al., 2013, McGinley et al., 2015b, Mineault et al., 2016, Niell and Stryker, 2010, Polack et al., 2013, Schneider et al., 2014, Vinck et al., 2015, Zhou et al., 2014).

Experiments have revealed strong effects of locomotion on V1 and A1 during baseline conditions, when there is no specific sensory stimulation. Some recordings suggest that locomotion is accompanied by depolarization. For instance, in the presence of a uniform gray screen, locomotion has been seen to depolarize V1 cells (Polack et al., 2013). However, its effect on spike output is unclear. Some studies reported that locomotion leaves the V1 baseline firing rate unaffected (Niell and Stryker, 2010), while others found that it increases firing rate in some V1 neurons and decreases it in others (Ayaz et al., 2013, Saleem et al., 2013, Vinck et al., 2015). Similarly, in the absence of externally delivered sounds, locomotion depolarizes A1 cells (McGinley et al., 2015a, Schneider et al., 2014) but does not affect their firing rate (Zhou et al., 2014).

Locomotion, however, appears to have opposite effects on the responses of areas V1 and A1 to sensory stimuli. In V1, locomotion increases the responses elicited by drifting grating stimuli, both in terms of firing rate (Ayaz et al., 2013, Niell and Stryker, 2010, Polack et al., 2013, Saleem et al., 2013, Vinck et al., 2015) and subthreshold depolarization (Polack et al., 2013). In A1, locomotion decreases the depolarization caused by auditory tones (Schneider et al., 2014, Zhou et al., 2014).

Given these results, it is not clear whether arousal has similar effects on different sensory areas and whether it affects sensory processing similarly across modalities. Moreover, for a wider view of the effects of locomotion on the sensory cortex, it would be useful to have data from the primary somatosensory area (S1). It is not known how locomotion affects S1 baseline activity and its response to individual sensory stimuli.

Here, we address these questions by using widefield imaging of mice expressing a voltage-sensitive fluorescence protein (VSFP) in excitatory neurons (Akemann et al., 2012, Carandini et al., 2015, Madisen et al., 2015). Using this technique, we estimated the local membrane potential simultaneously in large portions of cortex that include visual, auditory, somatosensory, and motor areas, while the head-fixed mouse was free to run on a treadmill.

## Results

To assess the effects of arousal simultaneously on multiple cortical areas, we imaged mice that expressed VSFP Butterfly 1.2 (Akemann et al., 2012, Madisen et al., 2015). The mice expressed VSFP in excitatory neurons, either in layer 2/3 (Rasgrf2-2A-dCre; Camk2a-tTA;Ai78, 12 mice) or in all layers (Emx1-Cre; Camk2a-tTA;Ai78, 6 mice). Signals from VSFP Butterfly 1.2 estimate local membrane potential in a region of cortex and are largely immune to the confounds of hemodynamic activity (Carandini et al., 2015).

**Figure 1:**
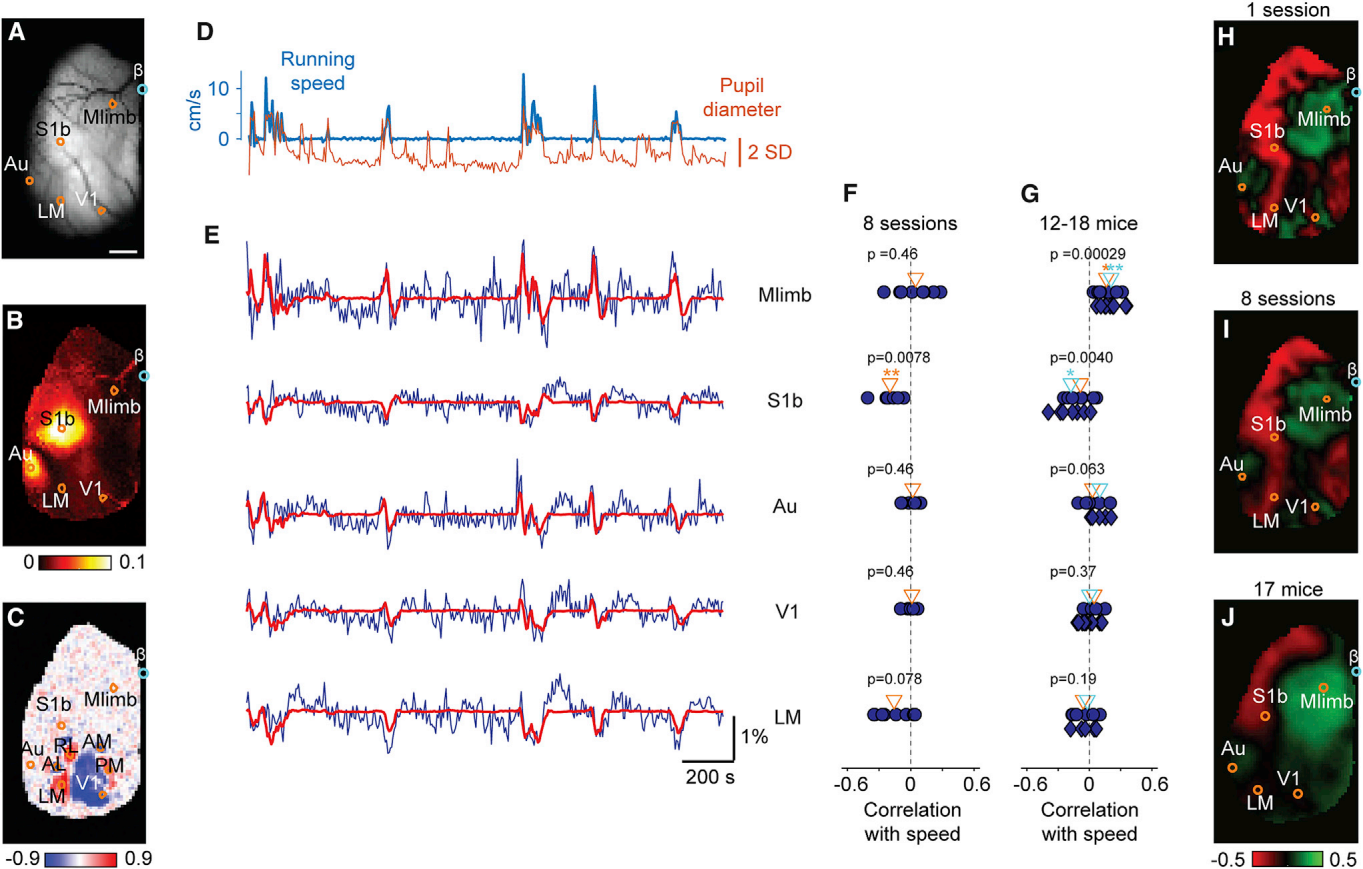
Effects of Locomotion on Estimated Membrane Potential across Cortical Areas (A) Imaging window over the left hemisphere of mouse cortex. The regions of interest (dots) are placed in limb sensorimotor cortex (Mlimb), barrel cortex (S1b), auditory cortex (Au), primary visual cortex (V1), and secondary visual area (LM). The scale bar represents 1 mm. (B) Air puffs to the whiskers elicit VSFP signals in S1b and Au. Heatmap indicates dR/R (percentage). (C) Visual areas revealed by computing maps of the visual field and measuring their sign. The sign indicates whether clockwise circles in the visual field map to clockwise (red) or anti-clockwise (blue) circles in cortex. (D) Running speed (blue) and pupil diameter (orange) measured over 30 min in an example imaging session. (E) The fluctuations in estimated membrane potential in the five regions of interest shown in (A), estimated by VSFP imaging (blue) and predicted (red) by filtering the running speed with temporal filters (shown in Figure 2A). (F) Correlation coefficient in 8 imaging sessions from the example mouse in (A)–(E). Triangle indicates mean across the sessions. Asterisks indicate significance (^∗^p < 0.05, ^∗∗^p < 0.01). (G) Correlation coefficient in all animals where we imaged motor and sensory areas (n = 17 for Mlimb, 14 for S1b, 12 for Au, 18 for V1, and 13 for LM). Symbols indicate individual animals (circles for Emx1-Cre mice and diamonds for Rasgrf-2A-dCre mice). Orange and cyan triangles indicate mean across Emx1-Cre and Rasgrf-2A-dCre crossed animals. (H) Map of the correlation coefficient between VSFP signal and running speed in the imaging session shown in (D) and (E). (I) Same as (H), averaged across the 8 imaging sessions in (F). (J) Same as (H), averaged across the 17 mice where the imaging window covered all the 5 areas in (G). Maps of correlation coefficient from different animals were aligned according to stereotaxic coordinates. Circles indicate average location of ROI across animals.

For each animal, we established the location of sensory and motor areas (Figures 1A–1C). We identified somatosensory barrel cortex (S1b; Figure 1B) and auditory cortex (Au; Figure 1B) by imaging responses to air puffs delivered to the contralateral whiskers (Figures 1A and 1B). We identified visual cortex based on the sign of the retinotopic mapping between the screen and the cortical surface (Figure 1C; Garrett et al., 2014, Sereno et al., 1994). This technique reliably located visual areas V1 and a secondary visual area (LM) in all mice and additional higher visual areas in some mice (Figure 1C). In addition to sensory areas, we used stereotaxic coordinates to select a region of interest in a sensorimotor region at the border between the primary sensory limb area and motor limb area. This region of interest, Mlimb, reveals the combined activity of these sensory and motor limb areas (Figure 1A).

### Effects of Arousal on Baseline Activity

Activity in all these cortical regions was markedly affected by arousal, as can be observed in a session in which a mouse ran for ∼10% of the time (Figures 1D and 1E). The mouse was free to run on a treadmill, and its running speed was clearly associated with arousal, because it increased with pupil dilation (Figure 1D). Running speed, moreover, had clear correlates in cortical activity, but these correlates were profoundly different in different parts of cortex (Figure 1E). Increases in running speed were associated with depolarizations in Mlimb and Au. Conversely, they were associated with hyperpolarization in S1b and LM.

These observations are consistent with measurements of correlation obtained across sessions and across mice (Figures 1F–1J). On average, Mlimb and Au tended to show positive correlation with running speed, and S1b tended to show negative correlation. The correlation pattern was not homogeneous across visual cortex; whereas V1 often showed weakly positive correlation, LM often showed mildly negative correlation.

These trends were in turn reflected in the maps of correlation (Figures 1H–1J). The results from a single example session showed correlations with running speed to be strongly positive around Mlimb and Au and strongly negative in a band passing through S1b and LM (Figure 1H). This basic structure was visible also after averaging across sessions (Figure 1I) and across mice (Figure 1J).

These simple measures of correlation, however, also revealed a source of variability across sessions and animals. Indeed, whereas the map of correlations for a single session showed marked differences across cortex (Figure 1H), these differences were weakened after averaging across sessions (Figure 1I) and across mice (Figure 1J). Accordingly, when pooling across the 8 sessions in the example mouse, correlations with running speed were significant at p = 0.05 (Wilcoxon signed-rank test) only in S1b (Figure 1F). Similarly, when pooling across the 12–18 mice (Figure 1G), correlations were significantly positive only in Mlimb (p = 0.00029, n = 17 mice) and significantly negative only in S1b (p = 0.0040, n = 14 mice).

We will see next that this variability across sessions and animals is not due to differences in brain function. Rather, it is explained by differences in the duration of running bouts across sessions and animals and by the fact that the relationship of voltage to running speed has a temporal structure.

### Dynamics of the Effects of Arousal

Inspection of the traces suggests that the effects of locomotion depend not only on cortical area but also on time following the onset of a bout (Figure 1E). For instance, Au and V1 were depolarized at the onset of locomotion, and this depolarization was often followed by hyperpolarization.

In these areas, a single measure of correlation that does not consider time could be misleading: correlation would appear positive for short bouts of running and closer to zero for long bouts. Sessions that differ in the prevalence of short or long bouts, in turn, would yield different values of correlation.

To capture these dynamics in the effects of arousal, we used a simple linear filtering model. In this model, the membrane potential $V_{A}\left(t\right)$ measured in area A is obtained by filtering the running speed of the animal r(t) with a temporal filter $f_{A}\left(\tau \right)$:

$$V_{A}\left(t\right)=\int f_{A}\left(\tau \right)r\left(t-\tau \right)d\tau \text{.}$$

For each area A, we estimated the filter $f_{A}\left(\tau \right)$ at each delay τ using L2-regularized linear regression.

The model provided a good account of the dynamics of the effects of locomotion (Figure 1E). Indeed, the traces predicted by the model in individual sessions (e.g., Figure 1E, red) captured the main features of the measured VSFP signals (Figure 1E, blue): biphasic effects of locomotion in V1 and Au and monophasic effects in Mlimb, S1b, and LM.

**Figure 2:**
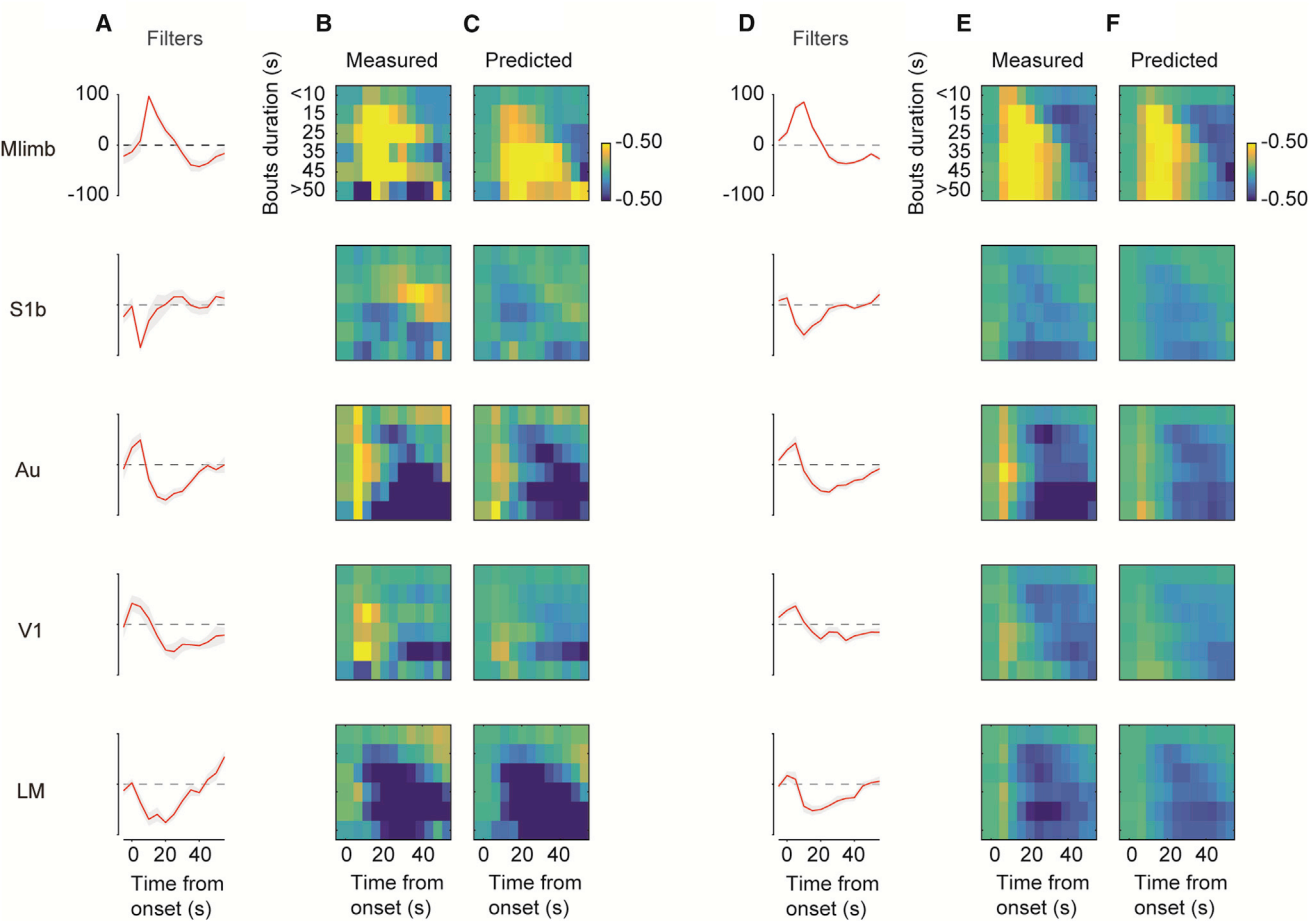
Time Dependence of the Effects of Locomotion on Estimated Membrane Potential (A) Filters estimated for the five cortical regions in the example mouse shown in Figure 1, showing the average filter (red) and SE (gray area) measured across 8 sessions in the same mouse. Individual filters are normalized by their absolute maximum amplitude. (B) Bout-triggered estimates of membrane potential. VSFP signals were aligned by running bout onset and averaged depending on bout duration. (C) Same as (B), for the VSFP signals predicted by filtering the running speed with the filters in (A). (D–F) Same as (A)–(C), for the results averaged across mice (n = 17 for Mlimb, 14 for S1b, 12 for Au, 18 for V1, and 13 for LM).

The temporal filters obtained from the model were consistent across sessions and different across cortical areas (Figures 2A and 2D). The filters were biphasic in Mlimb, Au, and V1, where they described depolarization followed by hyperpolarization. They instead were monophasic in the remaining areas, describing hyperpolarization in both S1b and LM (Figure 2A). Unlike the simple correlation values, the filters were highly consistent, as witnessed by the small error bars obtained when pooling across 8 imaging sessions from the same animal (Figure 2A) or across animals (Figure 2D). All filters were different from zero in their early phase (0–10 s, Wilcoxon signed-rank test, p < 0.01 in each area). They were also different from zero (p < 0.05) in the late phase (>10 s) in all areas except S1b.

To further explore the dynamics of the effects of arousal and to test the model’s performance, we studied the signals that followed locomotion onsets (Figures 2B, 2C, 2E, and 2F). We averaged activity aligned by bout onset and sorted by bout duration (Ma et al., 2016). As predicted by the model, this analysis revealed striking differences between cortical areas (Figures 2B and 2E). In some areas, locomotion had the same effect regardless of bout duration: steady depolarization in Mlimb and steady hyperpolarization in S1b and LM. Areas Au and V1 instead showed transient effects: depolarization at the onset of the bout followed by hyperpolarization. The same analysis gave similar results when applied to the model predictions (Figures 2C and 2F), further indicating that the filters faithfully describe the correlates of locomotion in different areas.

These differences in the effects of locomotion across cortical areas and across time were present in the estimated membrane potential but absent in the hemodynamic signals (Figures S1 and S2). Hemodynamic signals from changes in blood volume are measured during VSFP imaging by summing (instead of dividing) the signals from the two VSFP fluorophores (Carandini et al., 2015). These hemodynamic signals showed uniformly negative correlation with running speed, irrespective of cortical area (Figure S1) and time (Figure S2). This result indicates that locomotion is accompanied by widespread increases of blood volume, similar to pupil dilation (Pisauro et al., 2016).

Consistent with the view that locomotion is an assay for arousal, the effects of locomotion on voltage signals resembled the effects of pupil dilation (Figures S3 and S4). In the example animal (Figures 1A–1H), we measured pupil dilation in 2 of 8 sessions and found that it closely tracked the running speed. Accordingly, correlations of the voltage signal with pupil diameter (Figures S3A–S3H) were similar to those with running speed (Figures 1A–1I). Similar results were seen across animals (compare Figure 1J with Figure S3I). Likewise, the linear filters measured from pupil dilation (Figure S4) were similar to those measured from locomotion (Figure 2), and they provided even better predictions of the voltage traces (Figure S3D). Indeed, pupil dilation seems more informative, as it can reveal changes in arousal even when the animal is stationary.

### Effects of Arousal on Driven Sensory Activity

Having characterized the effects of locomotion on baseline activity, we turned to its effects on the processing of sensory stimuli. We begin with the responses to visual stimuli, and we focus on area V1, where the effects of locomotion have been the subject of substantial interest (Ayaz et al., 2013, Bennett et al., 2013, Fu et al., 2014, McGinley et al., 2015b, Niell and Stryker, 2010, Polack et al., 2013, Vinck et al., 2015).

**Figure 3:**
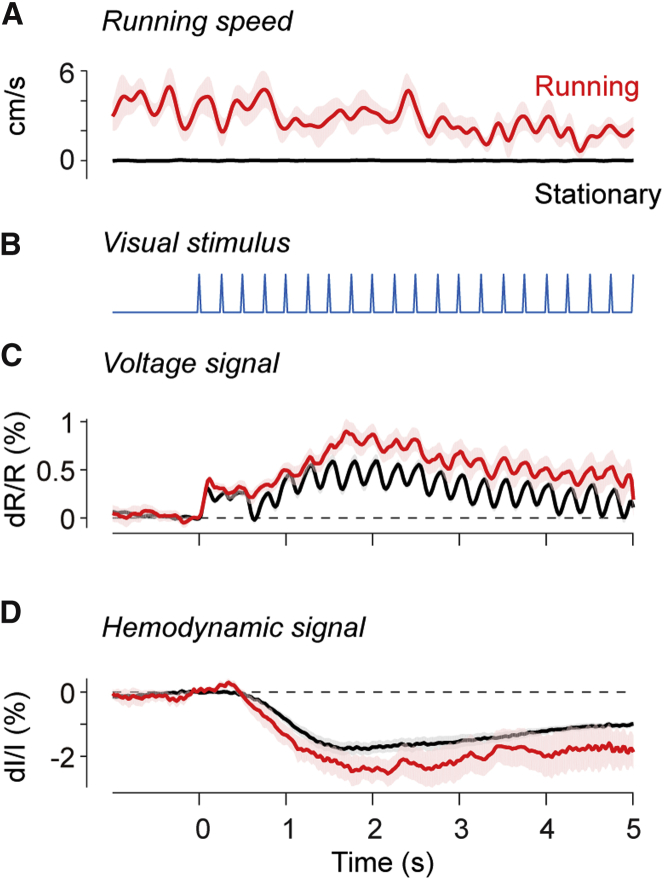
Running Speed, Voltage, and Hemodynamic Signals in V1 before and during Visual Stimulation (A) Average running speed for 10 mice imaged during contrast-reversing stimuli at 4 Hz, measured during locomotion (red) and in stationary periods (black). Red and gray shading areas indicate SE across animals. (B) Trace indicating the contrast reversals as a function of time. (C) Same format as (A), for the average VSFP signals measured in V1. Values measured before stimulus onset were subtracted from the traces. (D) Same format as (C), for the average hemodynamic signals measured in V1.

We delivered trains of visual stimuli and found that the resulting phasic responses were weaker during locomotion (Figures 3A–3C). Stimuli were flickering gratings presented in a vertical window to the side of the animal (50-degree eccentricity). During locomotion, mice did not generally change their running speed following the appearance of the stimuli (Wilcoxon signed-rank test, p = 0.3; Figures 3A and 3B). The stimuli caused both a sustained depolarization and a phasic response that oscillated at the frequency of contrast reversal (Figure 3C). Locomotion increased the sustained depolarization, but it decreased the phasic response (Figure 3C).

This reduction in the phasic responses during locomotion was absent in the hemodynamic signals, which varied over much slower timescales (Figure 3D). Indeed, the hemodynamic response estimated by summing the signals from a two VSFP fluorophores (Figure 3D) showed no sign of the phasic response: it was a much slower signal, with an initial lag of ∼0.4 s and a peak 1–2 s after stimulus onset (Pisauro et al., 2013).

**Figure 4:**
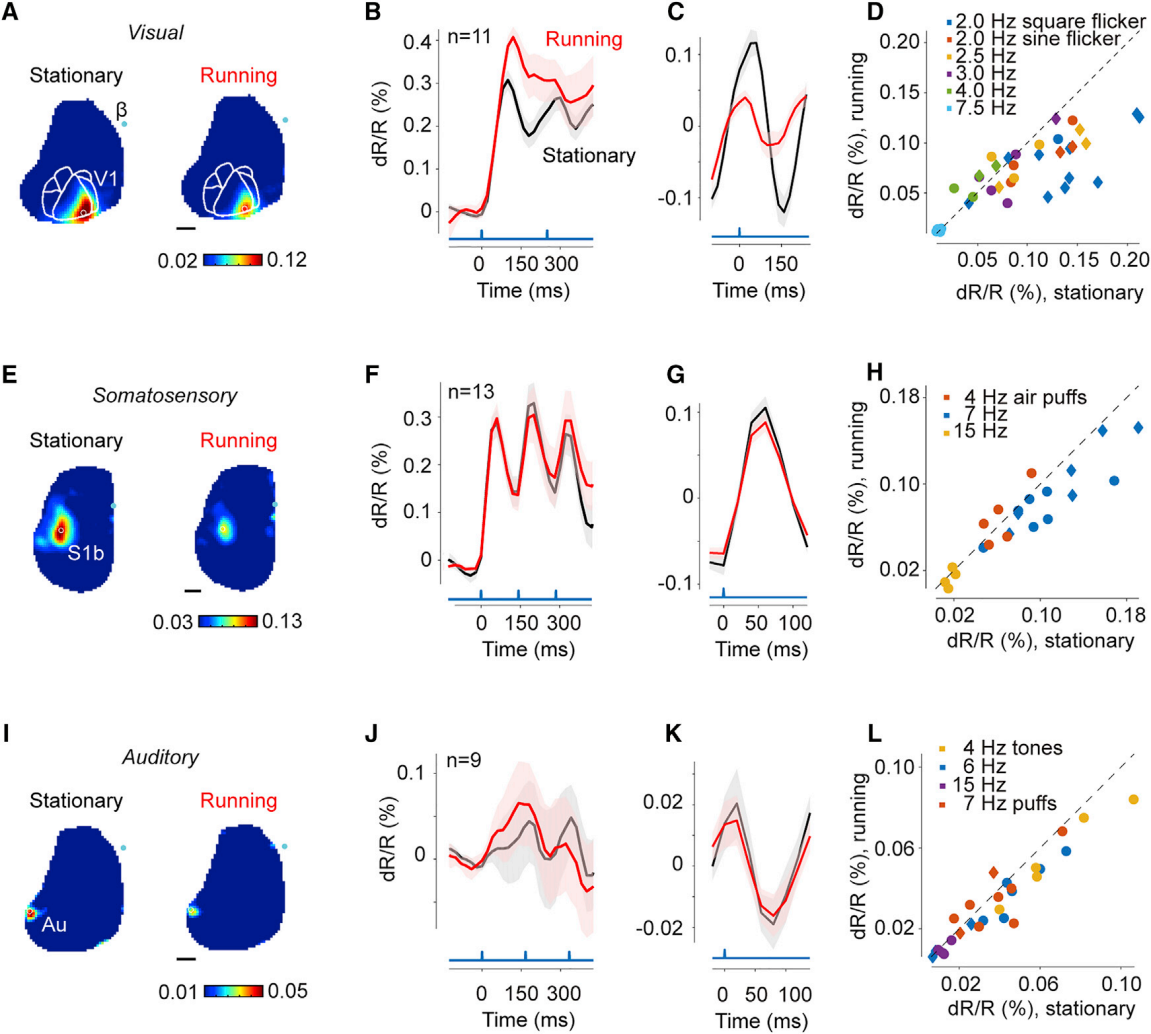
Effects of Locomotion on Phasic Responses to Sensory Stimuli in V1, S1b, and Au (A) The amplitude of the phasic response at the frequency of contrast reversal (4 Hz), imaged in an example session when the animal was stationary (left) or running (right). (B) Time traces of VSFP signal in V1 evoked by 4 Hz contrast-reversing grating placed at 50-degree eccentricity, averaged across animals (n = 11). The first 400 ms of responses are shown. Values on the y axis indicate deviation of VSFP signal from its average before the grating stimulus. Blue trace shows time of stimulus reversal. Red and gray shading areas indicate SE across animals. (C) Time traces of VSFP signal in V1 aligned by time of contrast reversal and averaged across the same animals as in (B). Values indicate deviation of VSFP signal from the average measured during stimulus presentation. Red and gray shading areas indicate SE across animals. (D) Amplitude of the phasic response from all the imaging sessions, where one sample represents one animal. Colors denote the stimulation frequency (circles for Emx1-Cre mice and diamonds for Rasgrf-2A-dCre mice). (E–H) Same as (A)–(D) for the VSFP signal evoked in S1b by trains of air puffs directed to contralateral whiskers. (I–L) Same as (A)–(D) for the VSFP signal evoked in Au by trains of tones. Red symbols in (L) indicate responses in Au to air-puff stimulation.

The reduction in phasic response caused by locomotion was a robust effect, seen across mice and across stimulation parameters (Figures 4A–4D). Phasic responses were typically strong in area V1 and much weaker in higher visual areas (Figure 4A). They began with a rapid depolarization that had similar onset regardless of locomotion (Figure 4B). The phasic response was an oscillation at the frequency of reversal, and the average cycle of this oscillation was markedly smaller during locomotion (Figure 4C). This effect was highly significant across the population; locomotion significantly reduced the amplitude of phasic responses (p = 0.0029 with 4 Hz stimulation [Figure 4C] and p = 0.00033 with all stimulation protocols [Figure 4D]).

Locomotion had a similar but weaker effect on somatosensory responses in S1b (Figures 4E–4H). To elicit responses in area S1b, we applied a train of air puffs to the contralateral whiskers (Madisen et al., 2015). These air puffs provided a strong drive to S1b (Figure 4E), eliciting a phasic response that oscillated at the frequency of stimulation (Figures 4F and 4G). The effects of locomotion in S1 were weaker than in V1 but pointed in the same direction; locomotion reduced the amplitude of the phasic response (Figures 4G and 4H). This reduction was consistent across mice (p = 0.00024 at 7 Hz stimulation [Figure 4G] and p = 0.0067 across all stimulus conditions [Figure 4H]).

Analogous effects were seen in Au (Figures 4I–4L). To elicit responses in Au, we delivered a train of tones (Madisen et al., 2015). This train of tones elicited a clear phasic response in Au (Figure 4I), which oscillated at the frequency of stimulation (Figures 4J and 4K). Once again, the effects of locomotion were weaker than in V1, but were similar: locomotion reduced the amplitude of the phasic response (Figures 4K and 4L). This reduction was small but significant (p = 0.0078 at 6 Hz stimulation [Figure 4K] and p = 0.00075 across all stimulus conditions [Figure 4L]).

The effects of running on the responses to sensory stimulation, therefore, show commonalities across sensory modalities. In all three sensory areas (primary visual, barrel, and auditory), locomotion reduced the phasic responses to individual stimuli in a train of stimuli.

## Discussion

We used widefield voltage imaging to assess the effects of arousal, assessed by locomotion on cortical activity, and we discovered that these effects can exhibit marked dependencies on time and are far from homogeneous across cortical areas. In the V1 and Au, locomotion first depolarized then hyperpolarized local baseline voltage, whereas in the S1b, it only hyperpolarized it. In all three areas, nonetheless, locomotion reduced the phasic voltage responses to trains of sensory stimuli.

When investigating these effects, widefield voltage imaging provides key advantages. First, it reveals activity simultaneously in multiple areas. This ability discounts uncontrolled factors that vary across experiments, such as the duration of running bouts, which may confound results when measuring activity in different areas in different experiments. Second, membrane potential variations do not necessarily affect firing rates. This feature is particularly important when measuring effects of locomotion, which may be largely subthreshold (Polack et al., 2013, Schneider et al., 2014). Finally, voltage imaging affords good temporal resolution, allowing one to follow both activity varying over hundreds of seconds and phasic sensory responses varying within a second (Akemann et al., 2012, Carandini et al., 2015, Madisen et al., 2015).

On the other hand, there are also clear limitations to the capabilities of widefield voltage imaging, particularly the voltage sensor VSFP-B1.2 used in this study. The sensor becomes nonlinear as membrane potential approaches 0 mV (Akemann et al., 2012). As for time constants, there appear to be two of them, one faster (2–6 ms) and one slower (20–100 ms). These timescales are >10 times faster than the ones we studied when measuring baseline activity, but they may interfere with measurements of a sustained component in sensory responses. Finally, our methods to estimate the voltage signal assume that this signal is uncorrelated with signals due to hemodynamics (Carandini et al., 2015), which is a simplification.

The results we obtained in visual and auditory areas provide a possible way to reconcile different views of the effects of locomotion on baseline activity. Some studies assert that locomotion depolarizes baseline membrane potential in both V1 (Polack et al., 2013) and A1 cells (McGinley et al., 2015a, Schneider et al., 2014) and increases baseline firing rate in V1 (Ayaz et al., 2013, Vinck et al., 2015). However, according to other studies, locomotion does not affect baseline firing rate either in V1 (Niell and Stryker, 2010) or in A1 (Zhou et al., 2014). By imaging Au and V1 simultaneously and studying the effects of locomotion as a function of time, we found that these effects are similar in the two areas: locomotion caused first a depolarization and then a hyperpolarization. The time dependence of these effects may explain discrepancies in the literature, as there could be variations in the duration of running bouts between animals in different studies.

Our data confirm that the effects of locomotion on area V1 depend on time (Vinck et al., 2015) and extend this observation to higher visual areas and other sensory areas. This time dependence indicates that simply measuring a correlation factor is not sufficient to describe the effects of locomotion. It can lead to different results in mice that run in shorter or longer bouts. Our study overcomes this difficulty by summarizing the effects of locomotion as temporal filters, which capture the time-dependent effect of locomotion. These filters explain how the correlation varies in the very same area depending on bout duration. Perhaps this reasoning might help explain why some studies found that locomotion depolarizes baseline membrane potential in V1 (Polack et al., 2013) and changes baseline firing rate (Ayaz et al., 2013, Saleem et al., 2013, Vinck et al., 2015), whereas others found no change in firing rate (Niell and Stryker, 2010).

We also found that locomotion had similar effects on the phasic responses of V1 and Au, which were smaller during locomotion than during stationary periods. This result agrees with earlier intracellular measurements, where locomotion was found to decrease the transient depolarization caused by auditory tones in A1 (Schneider et al., 2014, Zhou et al., 2014) and by periodic visual stimuli in V1 (Polack et al., 2013). It also appears consistent with the view that locomotion reduces response variability over time in both the auditory and visual cortex (McGinley et al., 2015a, Polack et al., 2013, Schneider et al., 2014). Other studies in V1 measured firing rate and found an increase in the responses elicited during locomotion (Ayaz et al., 2013, Erisken et al., 2014, Mineault et al., 2016, Niell and Stryker, 2010, Polack et al., 2013, Vinck et al., 2015). This discrepancy may be explained by the fact that firing rate depends not only on phasic depolarizations but also on baseline membrane potential. As we have seen, the latter is likely to be depolarized during locomotion.

Our findings also provide the first estimates of the effects of locomotion on the membrane potential of the S1b. We found that in the S1b, locomotion largely causes hyperpolarization. At first sight, this finding may seem to disagree with studies of the effects of whisking; intracellular measurements show the average membrane potential to be depolarized (Gentet et al., 2010, Poulet and Petersen, 2008) or unaffected by whisking (Poulet et al., 2012). However, mice in those studies were stationary, and results might have been different during locomotion.

We also found that locomotion reduced the phasic response of S1b to whisker stimulation. However, a more thorough study needs to be performed, because locomotion can be accompanied by whisking (Reimer et al., 2014), which decreases responses to passive whisker touch (Ferezou et al., 2007); in our study, we cannot distinguish these two effects. Moreover, air puffs are arguably not an ideal stimulus to study whisker activation: they are relatively uncontrolled, they can startle the animal, and they have an obvious auditory component.

We found that arousal caused varied effects on different cortical areas in terms of not only sign but also magnitude. A possible reason for this diversity may lie in differences in the relative weighing of inputs across the areas. Sources for arousal signals may include the thalamus, basal forebrain, and locus coeruleus (McGinley et al., 2015b). Different sensory areas of the cortex differ in the extent to which they receive input from these structures (Oh et al., 2014). While these mechanisms still need to be elucidated, our data indicate that arousal depolarizes some regions more than others and hyperpolarizes yet other regions, thus changing the overall configuration of the cortex.

## Experimental Procedures

Experimental procedures were conducted according to the UK Animals Scientific Procedures Act (1986), under personal and project licenses released by the Home Office following appropriate ethics review. Detailed description of the procedure is available in Supplemental Experimental Procedures.

We used mice expressing VSFP Butterfly 1.2 in excitatory neurons of layer 2/3 (Rasgrf2-2A-dCre;Camk2a-tTA; Ai78, n = 11) or in all layers (Emx1-Cre;Camk2a-tTA; Ai78, n = 7). We pooled the data from the Cre lines.

The 18 mice (10 males) were implanted with a head post and a thinned skull cranial window (Drew et al., 2010) at 1–10 months. After recovery, mice were head-fixed and imaged while freely moving on a treadmill. In some sessions, eye position and pupil dilation were captured by a charge-coupled device (CCD) camera.

We defined the onset and offset of locomotion as the time at which the treadmill motion signal crossed the threshold of 1 cm/s for at least 1 s (Polack et al., 2013). When analyzing sensory responses, trials with mean speed >1 cm/s were classified as “running” and the rest as “stationary.”

Visual stimuli were presented via LCD monitors (ProLite E1980, Iiyama) placed 19 cm from the animal. Air puffs (40 PSI) were delivered from a pressure injector (Pressure System IIe, Toohey Company) toward the whiskers via a silicone tube (0.5 mm diameter). Air puffs lasted 10–20 ms and were delivered in trains at 4, 7, or 15 Hz. Auditory stimuli were 13-kHz tones lasting 83 or 43 ms delivered in trains at 4, 6, or 15 Hz at 80 dB sound pressure level (SPL) via a magnetic speaker (Tucker-Davis Technologies) placed 19 cm from the animal.

We monitored VSFP signals with macroscope tandem lens system (Ratzlaff and Grinvald, 1991). As in previous reports (Carandini et al., 2015, Madisen et al., 2015), we imaged the two VSFP chromophores (mKate2 and mCitrine) via two sCMOS cameras (pco.edge, PCO). Images were acquired at 50–100 Hz, with a nominal resolution of 33 μm/pixel.

The trend in the recorded signals over the course of an imaging session (37 min on average) was removed with linear regression. The detrended signals were then analyzed by equalizing the gains at the heart beat frequency between the two cameras to estimate local membrane potential (Akemann et al., 2012, Carandini et al., 2015). The sum of the two signals captures large co-variations linked to the hemodynamic response (Carandini et al., 2015). To exclude possible residual contamination of hemodynamics in the voltage signal, the sum signal was filtered below 5 Hz, scaled by the regression coefficient with the voltage signal, and subtracted out from the voltage signal.

To investigate effect of locomotion on membrane potential, we calculated the Pearson correlation coefficient between imaging signals and locomotion speed and tested whether the correlation coefficients is deviated from 0 using the Wilcoxon rank-sum test. To quantify the phasic component of the sensory response, we calculated the oscillatory amplitude at the frequency of stimulation. To assess whether the phasic components depend on whether the animal is running or stationary, we used the Wilcoxon signed-rank test.

## Supplemental Information

Document S1. Supplemental Experimental Procedures and Figures S1–S4

Document S2. Article plus Supplemental Information
