# Effects of Arousal on Mouse Sensory Cortex Depend on Modality

**DOI:** 10.1016/j.celrep.2018.11.105

**Published:** 2018-12-11

**Authors:** Daisuke Shimaoka, Kenneth D. Harris, Matteo Carandini

(Cell Reports *22*, 3160–3167; March 20, 2018)

In the originally published version of this article, we wrote “…it would be useful to have data from the primary somatosensory area (S1). It is not known how locomotion affects S1 baseline activity.” However, two papers provided relevant measurements, Sofroniew et al. (2015) and Fisher et al. (2016). We have added these papers to the References and corrected the main text accordingly. The authors regret this error.

