# Supplementary material for: Effects of Arousal on Mouse Sensory Cortex Depend on Modality
Source: Cell Rep. 2018 Mar 20;22(12):3160–7. doi: 10.1016/j.celrep.2018.02.092 (PMC5883328; doi:10.1016/j.celrep.2018.02.092)
Supplement: Document S1. Supplemental Experimental Procedures and Figures S1–S4 [file mmc1.pdf]

Cell Reports, Volume *22*

Supplemental Information

Effects of Arousal on Mouse Sensory Cortex Depend on Modality

Daisuke Shimaoka, Kenneth D. Harris, and Matteo Carandini

Supplemental Experimental Procedures

Experimental procedures were conducted according to the UK Animals Scientific Procedures Act (1986), under personal and project licenses released by the Home Office following appropriate ethics review.

Transgenic lines

To obtain mice expressing voltage-sensitive fluorescent protein (VSFP) Butterfly 1.2 in selected neuronal populations, we crossed three lines of mice: (1) the Cre/tTA-dependent VSFP reporter line Ai78, Jax 023528 (Madisen et al., 2015); (2) a line expressing Tta in excitatory neurons, Camk2a-tTA line, Jax 007004; (3) a line expressing Cre in neurons of layer 2/3, Rasgrf2-2A-dCre or in neurons of all layers, Emx1-Cre, Jax 005628. The results of this crossing were mice expressing Butterfly 1.2 only in excitatory neurons of layer 2/3 (Rasgrf2-2A-dCre;Camk2a-tTA; Ai78, n = 11) or of all layers (Emx1-Cre;Camk2a-tTA; Ai78, n = 7). For inducing expression of transgene of VSFP in Rasgrf2-2A-dCre crossed animals, we administered Trimethoprim (TMP) diluted in 10% DMSO via oral gavage at 0.3mg/g body weight per day for consecutive 3 days.

We pooled the data from the Cre lines (Rasgrf vs. Emx1), because we did not find significant differences between the two, or did not have sufficient data to test for differences. When examining baseline activity, we found no significant difference between the two Cre lines in correlation between running speed and fluorescence, in all the five areas under consideration (Wilcoxon rank-sum test, Figure1**G**). For evoked activity, we compared phasic response amplitude between the Cre lines in each modality and stimulation frequency with the Wilcoxon rank-sum test. This test found only one possibly significant difference between, in Au stimulated at 6 Hz (p=0.095). In all the other 11 conditions, the test found no significant difference or was not applicable in some stimulation condition due to lack of samples.

Surgical procedure

The 18 mice (10 males) were implanted with a head post and a thinned skull cranial window (Drew et al., 2010) over the dorsal part of the cortex. An analgesic (Rimadyl) was administered on the day of the surgery (0.05 ml, s.c.), and on subsequent 2 days (in the diet). Anesthesia was obtained with isoflurane at 2-3% and kept 1-1.5% during surgery. To prevent dehydration during surgery, saline was administered every hour (0.01ml/g/h, i.p.). Body temperature was maintained at 37°C using a feedback-controlled heating pad, and the eyes were protected with ophthalmic gel. The head was shaved and disinfected with iodine, the cranium was exposed, the bone was thinned with a dental drill and a scalpel over the dorsal part of the cortex, and a metal head plate was secured with dental cement. The plate has a round opening (9 mm in diameter) for optical imaging. After the cement solidified, this opening was filled with transparent cement.

Treadmill

After recovery, mice were head-fixed and imaged while freely moving on a treadmill. The rotation of the treadmill was recorded and smoothed over a 1 s window. We defined the onset of locomotion as the time at which the treadmill motion signal crossed the threshold of 1 cm/s for at least 1 s. Similarly, we defined the offset of locomotion as the time at which the treadmill signal decreased below the threshold for at least 1 s (Polack et al., 2013). For the purposes of analyzing sensory responses, trials with mean speed > 1 cm/s were classified as "running", and the rest as "stationary". No effort was made to attenuate the sounds made by locomotion. While they ran, the animals often whisked. In some sessions, eye position and pupil dilation were captured by a CCD camera (DMK 21BU04.H, The Imaging Source), equipped with a macro zoom lens (MVL7000—18–108 mm EFL, f/2.5, Thorlabs). We extracted pupil diameter from gray-scale video frames by fitting an ellipse to the pupil image with custom software (github.com/carsen-stringer/FaceMap).

Sensory stimulation

Visual stimuli were presented via LCD monitors (ProLite E1980, Iiyama Corp.) placed 19 cm away from the animal. To identify areas V1 and LM (and occasionally, multiple other visual areas), we used one of 3 sets of stimuli: horizontal or vertical bars sweeping across the visual field (Garrett et al., 2014; Kalatsky and Stryker, 2003) (6 mice), brief flashes at 65 and 90 deg (Polack and Contreras, 2012) (6 mice) or flickering gratings (2 Hz flicker, i.e. 4 Hz contrast reversals), at various visual field positions (Carandini et al., 2015; Madisen et al., 2015) (6 mice). To probe visual responses, a standing

grating was placed in a vertical window (20 by 60 deg) with contrast reversing at 4, 5, 6, 8 or 15 Hz, centered at 50 deg eccentricity and 20 deg elevation.

Somatosensory stimuli were trains of air puffs delivered from a pressure injector (Pressure system IIe, Toohey Company) towards the bulk of the whiskers at a pressure of 40 PSI via a silicone tube (0.5 mm open tip diameter). Air puffs lasted 10-20 ms and were delivered in trains at 4, 7, or 15 Hz.

Auditory stimuli were 13 kHz tones lasting 83 or 43 ms delivered in trains at 4, 6, or 15 Hz, at 80 dB SPL via a magnetic speaker (Tucker-Davis Technologies) placed 19 cm away from the animal.

Stimulation with auditory and somatosensory stimuli, but not with visual stimuli, affected running speeds. When mice were running in the baseline condition they increased their running speed following delivery of auditory stimuli (Wilcoxon signed-rank test, p = 0.030) or of somatosensory stimuli (p = 0.0045). In the stationary condition, instead, we saw no significant effect of stimulus on running speed.

Imaging

We monitored VSFP signals with macroscope based on the tandem lens design and epi-illumination system (Ratzlaff and Grinvald, 1991), which we used in previous reports (Carandini et al., 2015; Madisen et al., 2015). Excitation light was provided by a blue LED (LEX2-B, Brainvision Inc.), through a band-pass filter at 482nm (FF01-482/35, Semrock Inc.) and a dichroic mirror (FF506-Di03, Semrock Inc.). VSFP FRET chromophores (mKate2 and mCitrine) were imaged via two sCMOS cameras (pco.edge, PCO AG). The first camera recorded the emitted fluorescence from mCitrine, which was reflected by a second dichroic mirror (FF593-Di03, Semrock Inc.), passed through an emission filter (FF01-543/50-25, Semrock Inc.). The second camera recorded the emitted fluorescence from mKate2, passed through the second dichroic mirror and an emission filter (BLP01-594R-25, Semrock Inc.). These cameras were controlled by an external TTL pulse synchronized with the sensory stimulation. The image acquisition rate was 50-100 Hz, with a nominal spatial resolution of 33 μm/pixel. Imaging sessions lasted 37 min on average.

Signal processing

The trend in the recorded signals over the course of recording period from the two cameras was removed with linear regression. The detrended signals were analyzed using the gain-equalization method (Akemann et al., 2012; Carandini et al., 2015), by equalizing the gains at the heart beat frequency between the two cameras. The gain equalization factors were obtained once per recording session at each pixel-basis, using the period when the animal was stationary. The sum of the two signals captures large co-variations linked to the hemodynamic response. The ratio of the two captures FRET signals linked to membrane potential variations (Carandini et al., 2015). To exclude possible residual contamination of hemodynamics in the ratiometric signal, the sum signal was filtered below 5 Hz then scaled by the regression coefficient with the ratiometric signal, and subtracted out from the ratiometric signal.

For the analysis of locomotion-evoked activity, we selected datasets when the mouse was running > 2% of the time. For the analysis of sensory-evoked responses, we used datasets when both running and stationary trials were recorded. We excluded a region of interest from the analysis if the oscillatory amplitude A(f) of the stationary trials at the stimulation frequency f was < 3*(A(f-1)+A(f+1)).

To investigate membrane potential activity during locomotion, we calculated the Pearson correlation coefficient between imaging signals and locomotion speed for each experiment. To this end, we linearly interpolated gaps between imaging sequences, which were typically 3 s, downsampled these signals at 0.2 Hz, and high-pass filtered at 0.005 Hz. We confirmed that the result did not depend on the interpolation methods. To test whether the correlation coefficients deviated from 0 we used the Wilcoxon rank-sum test, where one sample is the correlation coefficient of one recording session (Figure 1**F**; Suppl. Figure 1**E**; Suppl. Figure 3**E**) or one animal (Figure 1**G**; Suppl. Figure 1**F**; Suppl. Figure 3**F**)

To quantify the latency-dependence of the locomotion-evoked activity, we modeled the VSFP traces of each ROI by filtering the running speed with a temporal filter. In this model the membrane potential $V_A(t)$ measured in area A is obtained by filtering the running speed of the animal $r(t)$ with a temporal filter $f_A(\tau)$:

$$V_A(t) = \int f_A(\tau) r(t-\tau) d\tau$$

For each area A, we estimated the filter $f_A(\tau)$ at each delay τ using L2-regularized linear regression. To assess whether the temporal filter is deviated from 0 in latency dependent manner, we first averaged the filter values within early (0-10s) and late (>10s) windows, then applied Wilcoxon signed-rank test.

To quantify the phasic component of the sensory response, i.e. the average transient responses to the individual stimuli in the train, we calculated the oscillatory amplitude at the frequency of stimulation. To assess whether the phasic components depend on whether the animal is running or stationary, we used the Wilcoxon signed-rank test, where one sample is the average of the recording sessions from one animal.

To see if effects of locomotion depended on the specific Cre-line (Emx1 vs. Rasgrf), we first computed ratio of the phasic response amplitude between stationary and locomotion conditions in each animal, then compared the ratio between the two Cre lines using Wilcoxon rank-sum test, in each modality and stimulation frequency. The test found weak but significantly effect in Rasgrf compared to Emx1, in Au stimulated at 6Hz (p=0.095). In all the other conditions, the test found no significant difference or was not applicable in some stimulation condition due to lack of samples.

Region identification

We defined the cortical region corresponding to limb somatosensory/motor cortex (Mlimb) based on stereotaxic coordinates: 1.25 mm lateral and 0.5 mm posterior to bregma (Paxinos and Franklin, 2001; Zingg et al., 2014). To locate barrel cortex (S1b), auditory cortex (Au), and visual cortex (V1 and LM), instead, we used a functional method: we imaged the oscillatory response to 5 s trains of air puffs, auditory tones, and flickering gratings (Madisen et al., 2015). The location of the higher visual areas was functionally defined either by visual field sign (Garrett et al., 2014; Sereno et al., 1994), or peak response to flash grating (Polack and Contreras, 2012). We defined the region within 120 µm from these locations as Region of Interest (ROI) for each cortical area.

For each animal, we established the location of somatosensory and auditory cortex by imaging responses to air puffs delivered to the contralateral whiskers (Figure 1**A-B**). As shown previously (Madisen et al., 2015), these puffs reliably activated both barrel cortex (S1b, Figure 1**B**) and a region located more laterally, in auditory cortex (Au, Figure 1**B**). Judging from the Allen Brain Atlas, this region is unlikely to include the primary auditory area. Rather, it is likely to be a more dorsal or parietal secondary auditory area. It appears only partially in our image because our imaging window was placed close to horizontally. We confirmed that it was auditory by checking that it responded also to air puffs delivered to the ipsilateral whiskers and to tones delivered through a loudspeaker (Madisen et al., 2015).

We also identified visual cortex, by building maps of retinotopy (Figure 1**C)**. We built these maps by imaging responses to static or moving visual stimuli (Carandini et al., 2015; Kalatsky and Stryker, 2003; Polack and Contreras, 2012; Yang et al., 2007). We then parcellated the activated region into distinct visual areas by identifying changes in the sign of the retinotopic mapping between the screen and the cortical surface (Figure 1**C**). This sign is positive if clockwise circles in the visual field map to clockwise circles on cortex and negative if they map to anti-clockwise circles (Garrett et al., 2014; Sereno et al., 1994). Here we focus on visual areas V1 and LM. In some mice, we also identified higher visual areas RL, AL, AM, and PM (Figure 1**C**).

In addition to these sensory areas, we also selected a region of interest in a sensorimotor region corresponding to the limbs. We selected this region based on stereotaxic coordinates, at the border between the primary sensory limb area and motor limb area (0.5 mm posterior to bregma and 1.25 mm lateral, Zingg et al., 2014). This region of interest, Mlimb, reveals the combined activity of these sensory and motor limb areas (Figure 1**A).**

Supplementary Figures

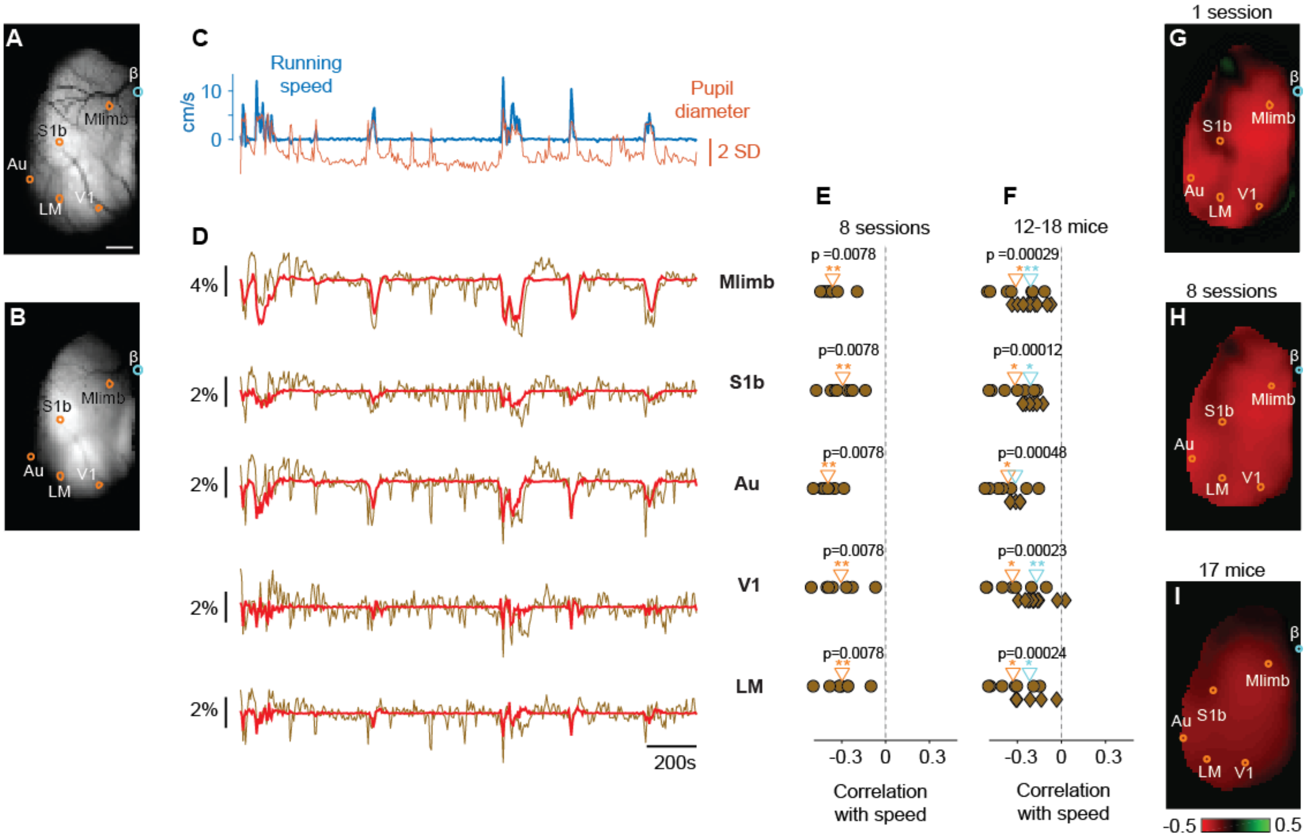

Suppl. Figure 1. **Effects of locomotion on hemodynamic activity across cortical areas**. Related to Figure 1.

A. Raw fluorescence signal of the donor channel showing Imaging window over the left hemisphere of mouse cortex. The regions of interest (*dots*) are placed in limb sensorimotor cortex (*Mlimb*), barrel cortex (*S1b*), auditory cortex (*Au*), primary visual cortex (*V1*), and secondary area (*LM*). The scale bar represents 1 mm.

B. raw fluorescence signal of the acceptor channel.

C. Running speed (*blue*) and pupil diameter (*orange*) measured over 30 min in an example imaging session.

D. The hemodynamic signals in the five regions of interest shown in **A**, estimated by imaging (*brown*) and predicted (*red*) by filtering the running speed with temporal filters.

E. Correlation coefficient in 8 imaging sessions from the example mouse in panels **A-D**. Triangle indicates mean across the sessions. Asterisks indicate significance (* p<0.05, ** p< 0.01).

F. Correlation coefficient in all animals where we imaged motor and sensory areas (n = 17 for Mlimb, 14 for S1b, 12 for Au, 18 for V1 and 13 for LM). Symbols indicate individual animals (*circles* for Emx1-Cre mice and *diamonds* for Rasgrf2-dCre mice). *Orange* and *cyan* triangles indicate mean across Emx1-Cre and Rasgrf2-dCre crossed animals.

G. Map of the correlation coefficient between the hemodynamic signal and running speed in the imaging session shown in panels **C** and **D**.

H. Same as panel **G**, averaged across the 8 imaging sessions in panel **E**.

I. Same as panel **H**. averaged across the 17 mice where the imaging window covered all the 5 areas in panel **F**. Maps of correlation coefficient from different animals were aligned according to stereotaxic coordinates. *Circles* indicate average location of ROI across animals.

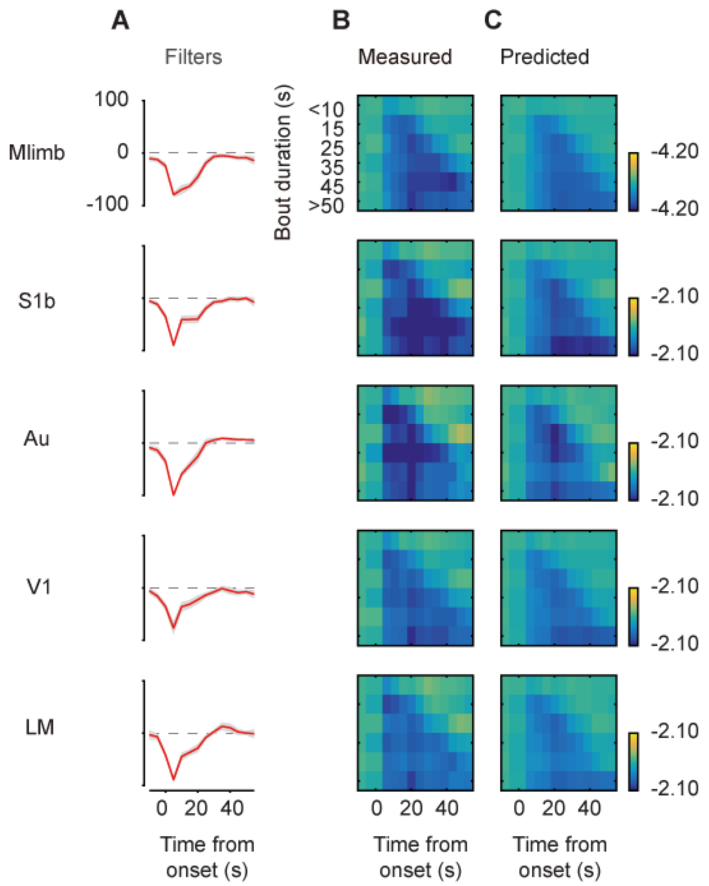

Suppl. Figure 2. **Time-dependence of the effects of locomotion on hemodynamic signals**. Related to Figure 2. Same format as Figure 2**D**-**F**, using running speed to predict hemodynamic signals instead of estimated membrane potential.

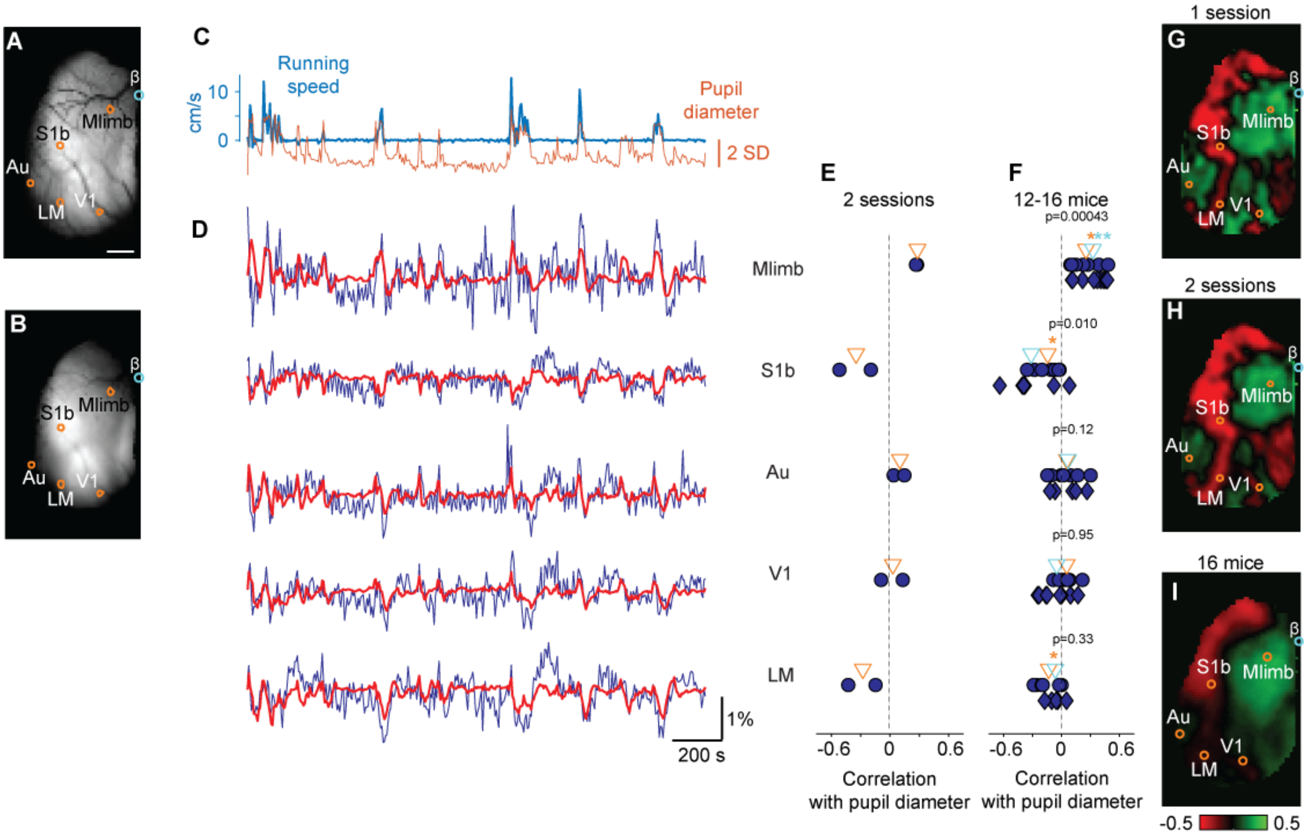

Suppl. Figure 3. **Effects of pupil dilation on estimated membrane potential across cortical areas**. Related to Figure 1.

B. raw fluorescence signal of the acceptor channel.

C. Running speed (*blue*) and pupil diameter (*orange*) measured over 30 min in an example imaging session.

D. The estimated membrane potential in the five regions of interest shown in **A**, estimated by VSFP imaging (*blue*) and predicted (*red*) by filtering the pupil diameter with temporal filters.

E. Correlation coefficient in 2 imaging sessions from the example mouse in panels **A-D**. Triangle indicates mean across the sessions. Asterisks indicate significance (* p<0.05, ** p< 0.01).

F. Correlation coefficient in all animals where we imaged motor and sensory areas (n = 16 for Mlimb, 14 for S1b, 12 for Au, 16 for V1 and 12 for LM). Symbols indicate individual animals (*circles* for Emx1-Cre mice and *diamonds* for Rasgrf2-dCre mice). *Orange* and *cyan* triangles indicate mean across Emx1-Cre and Rasgrf2-dCre crossed animals.

G. Map of the correlation coefficient between estimated membrane potential and pupil diameter in the imaging session shown in panels **C** and **D**.

H. Same as panel **G**, averaged across the 2 imaging sessions in panel **E**.

I. Same as panel **H**. averaged across the 16 mice where the imaging window covered all the 5 areas in panel **F**. Maps of correlation coefficient from different animals were aligned according to stereotaxic coordinates. *Circles* indicate average location of ROI across animals.

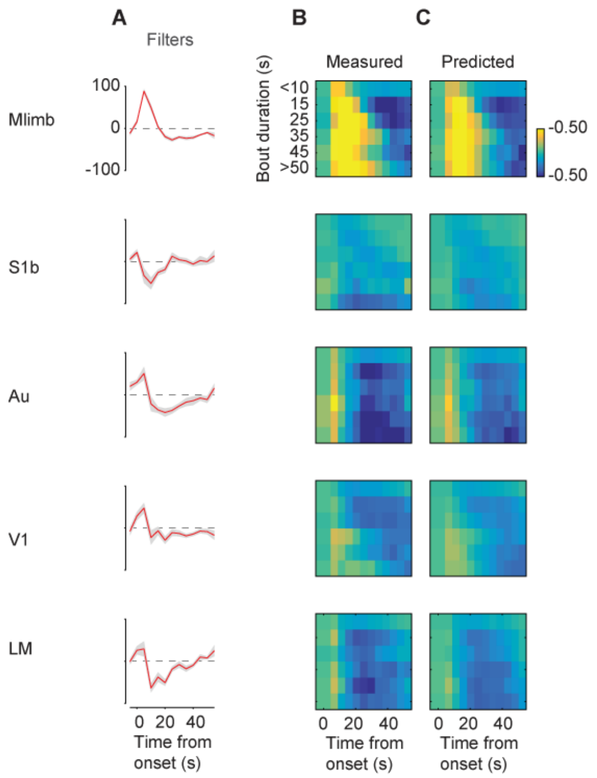

Suppl. Figure 4. **Time-dependence of the effects of pupil dilation on estimated membrane potential.** Related to Figure 2. Same format as Figure 2**D**-**F**, using pupil dilation as a regressor instead of running speed.
